# Supplementary material for: Health Professional Support for Friends and Family Members of Older People Discharged from Hospital After a Fracture: A Survey Study
Source: Geriatrics (Basel). 2025 Mar 7;10(2):36. doi: 10.3390/geriatrics10020036 (PMC11932223; doi:10.3390/geriatrics10020036)
Supplement: Supplementary file 1 [file geriatrics-10-00036-s001.zip › geriatrics-3504662-supplementary.pdf]

Supplementary File S1: The online survey

Version 1.0 – 19 June 2024

Section 1: Respondent Characteristics

Q1.1 Where do you work?

	YES
UK	
Europe (non-UK)	
North America	
Oceania	
South America	
Africa	
Asia	
Antarctica	

Q1.2 What is your principal clinical profession?

	YES
Physiotherapist	
Occupational Therapist	
Nurse	
Surgeon	
Physician	
Hospital manager	
Other	

Section 2: Carer Practices

Q2.1: Do you provide training, guidance or education to family members or friends who are going to support older people following bone fracture when at home?

	YES	NO
Response		

If YES to Q2.1:

Q2.2: Approximately what % of older people you treat following a bone fracture who go home do you provide family member/friend training, guidance or education to?

	%
Response	

Q2.3: Who provides this training, guidance or education in your service? *(tick all responses which apply)*

	Always	Sometimes	Never
Nursing team			
Physiotherapy Team			
Occupational Therapy Team			
General Multidisciplinary Team			
Social Workers			
Physicians			
Surgeons			
Other			

Q2.4: Which type of patients do carers get offered this training? (tick)

	Always	Sometimes	Never
Hip fracture			
Other lower limb fracture (non-hip)			
Spinal fracture			
Upper limb fracture			
Major trauma fracture			
Patients who live at home with identified carer			
Other			
Other			
Other			

Q2.5: Where are these provided?

	Always	Sometimes	Never
In hospital			
Out of hospital			
In and out of hospital			

Q2.6: How are these delivered? (tick)

	Always	Sometimes	Never
Face-to-face			
Virtual (telephone or online)			
Hybrid (face-to-face AND virtual)			

Q2.7: What is provided? (tick)

	Always	Sometimes	Never
Discussion on recovery expectations			
Practice manual handling (transfers/walking practice)			
Exercise prescription and progression advice			
Signposting to other post-discharge services			
Education on potential post-discharge complications			
Pacing and behaviour modification advice			

Advice on stress and anxiety management for caregivers			
Provision of contact details for further support			
Provision of written information/materials or online links			
Other:			

Q2.8: How long are these provided?

	Always	Sometimes	Never
Inpatient only			
Up to 2 weeks post-hospital discharge			
Up to 4 weeks post-hospital discharge			
Up to 6 weeks post-hospital discharge			
Longer than 6 weeks post-hospital discharge			

If NO to Q2.1:

Q2.9: If you could provide a carer training programme, for which types of older patients should this be offered to?

	Strongly Agree	Agree	Neutral	Disagree	Strongly Disagree
Patients living alone					
Patients living with an unpaid carer					
Patients living in a care home/care facility					
Patients following hip fracture					
Patients following lower limb fracture (non-hip)					
Patients following spinal fracture					
Patients following multiple fractures					
Major trauma patients					
Any patient who has experienced a fracture					
Other					
Other					

Section 3: Implementation

Q3.1: Could you implement a carer training programme into your clinical service today?

	YES	NO	POTENTIALLY
Response			

If Q3.1 is NO or POTENTIALLY

Q3.2: Would evidence from a clinical trial testing a carer training programme be valuable to help implement such a programme into your service?

	YES	NO	POTENTIALLY
Response			

If NO or POTENTIALLY or YES to Q3.1:

Q3.3: Is understanding the effectiveness of a carer training programme for older people discharged from hospital following bone fracture an important area of research?

	YES	NO	POTENTIALLY
Response			

Thank you for completing this survey

If you have any questions about this survey *OR* if you would like a copy of the results of this survey study, please contact Professor Toby
